# Study of Neuraminidase-Inhibiting Antibodies in Clinical Trials of Live Influenza Vaccines

**DOI:** 10.3390/antib9020020

**Published:** 2020-05-29

**Authors:** Yulia Desheva, Tatiana Smolonogina, Svetlana Donina, Larisa Rudenko

**Affiliations:** Federal State Budgetary Scientific Institution “Institute of Experimental Medicine”, Saint Petersburg 197376, Russia; smolonogina@mail.ru (T.S.); sveta.donina@gmail.com (S.D.); vaccine@mail.ru (L.R.)

**Keywords:** live influenza vaccine, immunogenicity, neuraminidase inhibiting antibody

## Abstract

Background: Currently, the immunogenicity of influenza vaccines is assessed by detecting an increase of hemagglutination inhibition (HI) antibodies. As neuraminidase (NA)-based immunity may be significant in protecting against influenza infection, detection of neuraminidase inhibiting (NI) antibodies may improve the assessment of the immunogenicity of influenza vaccines. Methods: We investigated the immune response to NA in people after immunization with live influenza vaccines (LAIVs). A number of A/H7NX or A/H6NX viruses were used to detect NI antibodies, using an enzyme-linked lectin assay (ELLA). Results: Seasonal LAIV immunization stimulated an increase in NI antibodies not only to homologous A/H1N1 influenza, but also to A/H1N1pdm09 and A/H5N1 influenza. After A/17/California/09/38 (H1N1) pdm09 LAIV vaccination, there was no statistical relationship between post-vaccinated antibody seroconversion and two surface glycoproteins in serum samples obtained from the same individuals (*p* = 0.24). Vaccination with LAIV of H5N2, H2N2, H7N3, and H7N9 subtypes led to 7%–29.6% NI antibody seroconversions in the absence of HI antibody conversions. There was relatively low coordination of hemagglutinin (HA) and NA antibody responses (r = 0.24–0.59). Conclusions: The previously noted autonomy for HI and NI immune responses was confirmed when assessing the immunogenicity of LAIVs. Combining the traditional HI test with the detection of NI antibodies can provide a more complete assessment of LAIV immunogenicity.

## 1. Introduction

The determination of antibodies that inhibit neuraminidase (NA) in the influenza virus (the genus *Influenzavirus*, the family *Orthomyxoviridae*) is of interest to science and for clinical practice. It can be used when examining the levels of collective immunity to new influenza viruses, and for assessing the immunogenicity of influenza vaccines [1]. Currently, the immunogenicity of influenza vaccines is assessed by detecting an increase of hemagglutination inhibition (HI) antibodies. As NA-based immunity may be significant in protection against a new antigenic variant of an influenza virus, detection of neuraminidase inhibiting (NI) antibodies may improve the study of the immunogenicity of newly developed influenza vaccines. In the present study, we investigated the formation of NI antibodies in people after immunization with live influenza vaccines (LAIVs) of pandemic and potentially pandemic subtypes. The World Health Organization has noted that it is important to develop new, or improve and standardize existing, methods for detecting the humoral immune response to NAs. NI antibodies are effective in limiting the spread, reducing the severity, or preventing the development of transferred diseases [1]. However, a lack of experimental and clinical data in this specific area makes it difficult to quantify the protective efficacy of these antibodies and, therefore, to develop criteria for the immunogenicity of influenza vaccine assessment based on the evaluation of NI antibodies. To obtain reassortant influenza A vaccine viruses, a cold-adapted “master donor strain” (MDS) A/Leningrad/134/17/57 (H2N2) is currently used [2]. For the production of type B vaccine strains, MDS B/USSR/60/69 [3] is used. The seasonal vaccine viruses A/H1N1, A/H3N2 and B have a 6:2 genome composition; that is, they acquire six genes of internal and non-structural proteins from MDS, and two genes of surface glycosylated proteins— hemagglutitin (HA) and NA—from epidemic influenza viruses.

LAIVs have been used in Russia since the 1960s and were licensed for use in North America and Europe in 2003 [4]. Recently, Russian LAIVs were licensed in China (http://www.biodiem.com/wp-content/uploads/2020/02/20-02-28-LAIV-influenza-vaccine-approved-in-China.pdf). The efficacy of intranasal LAIVs is up to 80% in children under six years of age, and 40% in adults [5,6].

As seasonal influenza vaccines cannot protect against infection with avian influenza viruses, the World Health Organization (WHO) carries out monitoring and analysis of potentially pandemic zoonotic influenza viruses in the development of appropriate vaccines [7].

In the current study, we used the enzyme-linked antibody assay (ELLA) to study the formation of antibodies to NA of pandemic and potentially pandemic influenza viruses after LAIV immunization.

## 2. Materials and Methods

### 2.1. Ethics Statement

This study involved a retrospective analysis of serum samples from clinical trials of pandemic and potentially pandemic LAIVs based on A/Leningrad/134/17/57 (H2N2) MDS (Table 1).

The genome composition of almost all vaccine strains was 6:2, except A/17/duck/Potsdam/86/92 (H5N2), which had a 7:1 genome composition; the NA gene of A/17/duck/Potsdam/86/92 (H5N2) was inherited from A/Leningrad/134/17/57 (H2N2) MDS.

We also investigated sera obtained from volunteers who were 18–20 years old in the fall of 2005, before and 21 days after vaccination with seasonal trivalent live influenza vaccine (LAIV) 2005–2006. The trivalent vaccine was manufactured by “Microgen” (Irkutsk, Russia) and contained A/17/New Caledonia/99/145(H1N1), A/17/California/04/06(H3N2), and B/60/Jilin/01/1 (B/Yamagata-like) [17].

Additionally, we tested the archive serum samples collected in the fall of 2010 and 2011 from routine tests, as well as the sera from patients of the Institute for Experimental Medicine Medical Research Center collected in the 2016, and the paired sera from influenza convalescents provided by the Institute of Influenza. The patients were fully informed of the research procedures and any risks associated with participation, and consented to participate in these scientific projects. None of the authors collected any of the samples used in the study, or had access to the confidential information of patients; data were anonymized before being made available to the authors. The sera were stored frozen at −20 °C until testing. Following obtaining the approval from the Local Ethics Committee in the Institute of Experimental Medicine, No. 3/17 of 30 November 2017, these sera were provided for serological tests.

### 2.2. Viruses

To evaluate serum NI antibodies using the enzyme-linked lectin test (ELLA), we developed a number of A/H7NX or A/H6NX viruses (Table 2).

To detect NI antibodies after vaccination with LAIVs based on the A/17/mallard/Netherlands/00/95 (H7N3) vaccine strain, we used the A/tern/South Africa/61 (H5N3) influenza virus.

All viruses were cultured in the allantoic cavity of 10-day-old developing chicken embryos (CEs), which were provided by the Sinyavino poultry farm (Kirovsk Area, Leningrad Region, Russia), at 33 °C for 48 h. For ELLA, the viruses were purified and concentrated on a stepwise 30%/60% sucrose gradient, then aliquoted, and stored at −70 °C.

### 2.3. Serology

#### 2.3.1. Enzyme-linked Lectin Assay (ELLA)

We evaluated NI antibodies using ELLA as previously described [18]. Briefly, 96-well plates (Greiner Bio-One, Kremsmünster, Austria) were coated overnight with 150 μL of 50 μg/mL fetuin (Sigma-Aldrich, St. Louis, MO, USA). Then, 60 μL of sera was heated at 56 °C for 30′, serially diluted in phosphate buffered saline with bovine serum albumin (PBS-BSA), and incubated with an equal volume of pre-diluted virus for 30′ at 37 °C. After incubation, 100 μL of the mixture was layered on the fetuin-coated wells. After 1 h of incubation at 37 °C, the plates were rinsed, and NA activity was estimated by incubating with peroxidase-labeled peanut lectin (2.5 μg/mL, Sigma-Aldrich, St. Louis, MO, USA) for 1 h at room temperature, followed by washing and then adding 100 μL of peroxidase substrate 3,3’,5,5’-Tetramethylbenzidine (TMB). The reaction was terminated after 5 min with 100 μL of 1 N sulfuric acid. The optical density (OD) values were measured at 450 nm using the universal microplate reader (ELx800, Bio-Tek Instruments Inc., Winooski, VT, USA). The titer of serum NI antibodies was determined as the reciprocal dilution of the sample with 50% inhibition of NA activity. A twofold increase in the titer of NI antibodies after vaccination was considered significant.

#### 2.3.2. Hemagglutination Inhibition Test

The HI test with blood sera was carried out using a 0.75% suspension of human red blood cells (Group “0”) in “U”-bottom 96-well polymer plates, using previously published procedures [19]. The investigated sera were treated with receptor destroying enzyme (RDE) which is an extract of Vibrio cholerae NA (Denka Seiken Co., Tokyo, Japan) and heated at 56 °C for 30 min, and then tested in duplicate for HI antibodies with the test vaccine antigens starting from 1:5 or 1:10 initial serum dilutions. The titers of HI antibodies were expressed as the reciprocal of the highest serum dilution at which HI was observed. A fourfold increase in the titer of HI antibodies after vaccination was considered significant.

#### 2.3.3. Microneutralization Test

Determination of neutralizing antibodies was carried out using the microneutralization (MN) test in Madin–Darby canine kidney (MDCK) cells with no-RDE-treated sera, as previously described [19].

### 2.4. Statistical Analysis

Data were analyzed using Statistica software, version 6.0 (StatSoft Inc., Tulsa, OK, USA). Antibody levels were presented as geometric mean titers (GMTs). For statistical analysis, antibody titers were expressed as log2 inverse final dilutions. Comparisons of two independent groups were carried out using the non-parametric Kolmogorov–Smirnov two-sample test. To compare several independent groups, we used the Kruskal–Wallis analysis of variance (ANOVA), followed by multiple pairwise comparisons based on the Kruskal–Wallis sums of ranks. Comparisons of two dependent variables were performed using the Wilcoxon matched pairs test. Fisher’s exact two-tailed test was carried out in the case of nominal variables. A nonparametric measure of the statistical relationship between the two variables was performed using the Spearman rank correlation coefficient (r). A *p*-value < 0.05 was considered to be statistically significant.

## 3. Results

Table 3 presents antibody levels (GMTs) against both main influenza glycoproteins—HA and NA—detected before vaccination and after revaccination with LAIVs developed against pandemic and potentially pandemic influenza viruses.

According to the HI test and ELLA, the initial immunological status of the vaccine and placebo groups of volunteers did not differ (Mann–Whitney U test, *p* > 0.05). Pre-vaccination HI antibody titers to new vaccine strains did not exceed the lower detection limits of the HI method, which was determined by the initial serum dilution (1:5–1:10). Revaccination was carried out 21 or 28 days after the first vaccination; the same period after revaccination, blood serum was collected [9,10,11,13,14,16]. The HI antibody rises after revaccination were 1.6–3.4 (Table 3); this increase was statistically significant in all cases compared with pre-vaccination HI levels.

The levels of NI antibodies against the NA of different subtypes were variable (Table 3). The highest antibody levels before vaccination were detected for the NA of N1 and N2 subtypes; that is, for viruses that have been in circulation for a long time. At the same time, the level of antibodies in the A/H5N2 vaccine strain’s NA, which was derived from a donor strain of A/Leningrad/134/17/57 (H2N2), was low. Differences in anti-N2 antibody titers for A/H5N2 and A/H2N2 before vaccination can be explained by the fact that, between 1957 and 1968, the NA of A/H2N2 viruses acquired 21 amino acid substitutions in the enzymatically active and antigenic regions of the globular head [20]. No antibodies were detected before vaccination against N9. The increase in geometric mean titers of NI antibodies after vaccination was 1.2–2.4. Like the increase in HI antibodies, the increase in antibodies to the NA of vaccine strains was statistically significant (Table 3). In all studies, there was no increase in either the HI or NI antibodies in the placebo group.

In order to study the levels of pre-existing antibodies for potentially pandemic and pandemic viruses, we examined extended groups of unvaccinated volunteers for the presence of NI antibodies, including the N1 of the avian influenza virus A/Vietnam/1203/04 (H5N1) and the drift variant of the pandemic virus A(H1N1)pdm09—the A/South Africa/3626/2013 (H1N1)pdm09 influenza virus (Table 4).

The lowest preexisting NI antibody levels were revealed against A/Anhui/1/13(H7N9) and A/South Africa/3626/13(H1N1)pdm09 influenza viruses (0% and 7% of persons with NI antibody titers > 1:20, respectively).

Despite the fact that the A/mallard/NL/12/00(H7N3) virus was never in the circulation, pre-existing antibodies to the NA of the N3 subtype were detected.

Interestingly, before the start of A/H1N1pdm09 circulation, only 7.1% of examined persons had A/California/7/09(H1N1)pdm09-specific NI antibodies, which obviously were cross-reacting antibodies caused by contacts with previously circulating A/H1N1 strains. However, after the pandemic virus entered circulation, the titers of NI antibodies increased not only against A/H1N1pdm09, but also for the NA of the A/Vietnam/1203/04(H5N1) avian influenza virus. In 2011, 46% of the examined volunteers had cross-reacting NI antibodies to A/Vietnam/1203/04(H5N1), compared with 16% in 2005 (*p* < 0.05). These data confirm previously obtained information on the cross-reaction of N1-directed antibodies acquired as a result of infection and vaccination with inactivated vaccines [21,22]. In 2016, we assessed “herd” immunity to the A/South Africa/3626/13(H1N1)pdm09 NA, as this drift variant of A/H1N1pdm09 had been recommended for inclusion in the composition of influenza vaccines in the 2016–2017 influenza season (https://www.who.int/influenza/vaccines/virus/recommendations/summary_a_h1n1pdm09_cvv_nh1617.pdf). From 2009, A/California/7/09(H1N1)pdm09-like viruses circulated, which, from 2010 to 2016, were consistently included in vaccines. Not surprisingly, the level of collective immunity to A/California/7/09(H1N1)pdm09 was, by 2016, significantly higher than that to A/South Africa/3626/13(H1N1)pdm09. It was shown that, in the 2015–2016 epidemic season, the levels of NI antibodies ≥1:40 to A/California/7/09(H1N1)pdm09 amounted to about 30% [23].

A higher proportion of persons with pre-existing NI antibodies against A/H2N2 1966 (year of isolation) was found among elder volunteers compared with younger people (*p* < 0.05). This confirms the previously obtained data on the age-related differences in the levels of pre-existing antibodies to NA, which may be associated with first contacts with influenza viruses [24].

**Figure 1:**
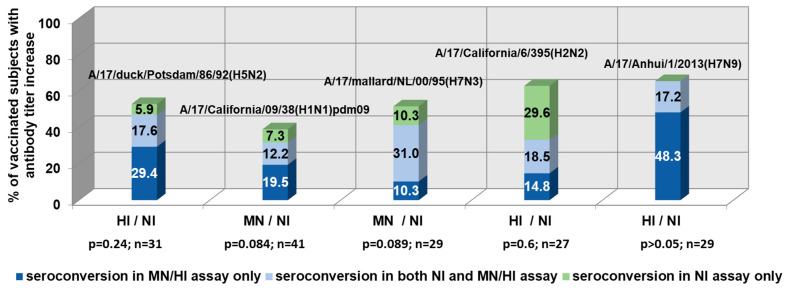
The coincidence of neuraminidase-inhibiting (NI) and hemagglutination/microneutralization (HI/MN) antibody seroconversions in the paired sera of live influenza vaccine (LAIV)-vaccinated subjects (≥twofold increase in NI titers; ≥fourfold increase in HI/MN titers).

Figure 1 demonstrates the NI and HI/MN antibody seroconversions in the paired sera of LAIV vaccinated subjects. From 6% to 29.6% of volunteers vaccinated with LAIVs showed a significant increase in serum NI antibody titers in the absence of HI/MN antibody conversions.

There has been relatively low coordination of HA and NA antibody responses (r = 0.24–0.59) in clinical studies of LAIVs. These data confirm the previously obtained data on NI antibodies as independent indicators of influenza immunity, other than hemagglutination inhibiting antibodies [25]. In the case of immunization with the A/17/Anhui/2013/61 (H7N9) LAIV, there was no increase in NI without the development of an antibody response to HA, despite the absence of anti-N9 antibodies before vaccination. Thus, in this case, the HA acted as an immunodominant antigen.

**Figure 2:**
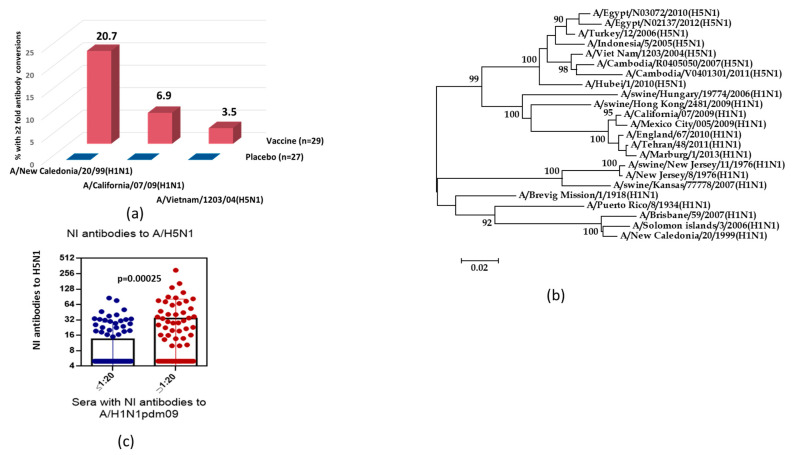
Cross-reactivity of NI antibodies against N1. (**a**) The twofold seroconversions of NI antibody in 18–20 years old volunteers after immunization with seasonal trivalent LAIV (2005–2006 flu season). (**b**) Phylogenetic tree of the N1 neuraminidase of influenza virus isolates belonging to different HA subtypes. (**c**) NI serum antibody titers to A/Vietnam/1203/04(H5N1) in the sera with A/California/7/09(H1N1)pdm09 NI titers ≤1:20 (*n* = 82) and NI titers >1:20 (*n* = 64).

In the next part of the study, we analyzed the cross-reactive properties of antibodies after immunization with seasonal trivalent LAIV. Figure 2a demonstrates that immunization of volunteers who were 18–20 years old with seasonal LAIV (2005 year of formulation) containing the A/New Caledonia/20/99 (H1N1) vaccine strain stimulated an increase in NI antibodies, not only to homologous seasonal A/H1N1 influenza virus, but also to the antigenically distinct variants A/California/7/09 (H1N1)pdm09 and A/Vietnam/1203/04(H5N1). Antibody levels to the A/New Caledonia/20/99 (H1N1) influenza virus to some extent correlated with titers of the pandemic strain A/H1N1pdm09 (rs = 0.60; *n* = 56; *p* < 0.001) and avian A/H5N1 influenza virus (rs = 0.46; *n* = 56; *p* < 0.001).

The phylogenetic analysis of NA amino acid sequences of avian A/H5N1 and epidemic and pandemic A/H1N1pdm09 influenza strains demonstrated the higher homology between amino acid sequences of H5N1/2004 and H1N1/pdm09 NA than between NAs of avian A/H5N1 and epidemic A/H1N1 viruses, which had circulated before 2008 (87.9% vs. 82.6%) (Figure 2b). Figure 2c shows the titers of NI antibodies to the H5N1/2004 virus among volunteers examined in 2011; that is, after the pandemic A/H1N1pdm09 influenza virus entered circulation. The mean NI anti-H5N1/2004 antibody titer among subjects who were positive to H1N1/2009pdm, according to NI assay, was 4.5-fold higher than that among seronegative volunteers.

**Figure 3:**
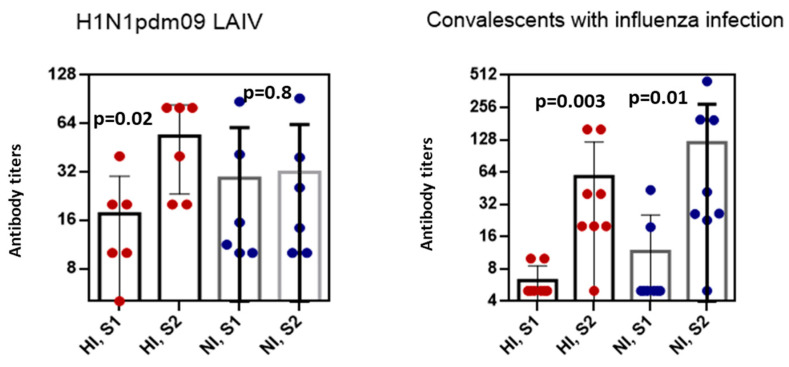
The HI and NI antibody titers to A/California/07/2009(H1N1)pdm09 after vaccination with LAIV or natural infection in persons with ≥fourfold HI antibody conversions. Antibody titers in LAIV recipients before vaccination and on day 21 after vaccination (*n* = 6). Antibody titers in the paired sera collected from convalescents with laboratory confirmed influenza A (*n* = 8).

To compare the levels of NI antibodies after LAIV vaccination and infection, we analyzed the NI antibody content in patients with confirmed influenza A infection, and recipients of A/17/California/09/38 (H1N1)pdm09 LAIV with fourfold or more HI antibody rises. It was shown that on day 6–8 after onset of infection, the levels of NI antibodies were significantly higher than at the beginning of the disease (*p* = 0.01). Post-vaccination titers of HI antibodies after administration of A/17/California/09/38(H1N1)pdm09 LAIV were only slightly higher than pre-vaccination titers (Figure 3).

Fisher’s exact test revealed no statistical relationship between post-vaccinated antibody seroconversion and two surface glycoproteins of the H1N1/2009pdm09 virus in serum samples obtained from the same individuals after vaccination (*p* = 0.24), although after infection, this ratio was statistically significant (*p* < 0.0001).

## 4. Discussion

LAIVs against seasonal influenza are harmless and cause persistent homologous responses to wild-type influenza viruses [4]. Owing to the high variability of influenza HA, each vaccine formulation should be updated almost annually. Although licensed influenza vaccines are primarily designed to provide homotypic protection, studies show that immunization with influenza vaccines or natural infections with seasonal influenza A strains induce serum cross-reactive antibodies to antigenically different variants of viruses [26,27]. Traditionally, the assessment of the immunogenicity of influenza vaccines is carried out by assessing the level of serum antibodies to the main antigenic component of the virus, the HA. The study of antibodies to influenza NA is useful when influenza viruses with new HAs appear in circulation, and the population is not immune to these subtypes. Enzyme-linked lectin assay is the simplest and least costly method of determining NA-inhibiting antibodies. To avoid non-specific reactions with antibodies to influenza A, viruses of subtype H7 and H6 are used as a source of HA [28].

The protective role of serum antibodies to the NA of influenza viruses was discovered in the 1970s. During the outbreak of Hong Kong influenza in 1968, the frequency of infection with the A/H3N2 virus and the number of patients with severe clinical symptoms were inversely related to the level of antibodies to N2, which was estimated even before the start of the circulation of the new subtype strain [29]. Later, it was found that NA antibodies reduce the severity of influenza infection in humans [30]. In animal experiments, it was demonstrated that double immunization of NA in the form of a purified or recombinant antigen, followed by infection with a drift variant of a virus with an NA of the same subtype, reduced viral reproduction in the lungs of mice by up to 1000 times, preventing deaths [31].

In 2009, the WHO announced the emergence of a new influenza pandemic caused by the influenza A/H1N1pdm09 virus, which had not previously been isolated from humans or animals [32]. Unlike previous seasonal epidemics, an unusually severe disease was often observed among young and middle-aged people, while older people suffered the pandemic relatively easily. In this regard, immediately after the announcement of the pandemic, studies of the immune status against influenza viruses in various populations began [33]. It was revealed that, even a year after the spread of the pandemic strain, most people did not have HI antibodies to the A/H1N1pdm09 virus at “protective” levels of ≥1:40. At the same time, a number of studies have shown that some people have cross-reacting antibodies to the NA of influenza viruses with new HA subtypes, resulting from contacts with previously circulating variants [22,34]. This may be significant in reducing mortality and limiting the spread of the virus in the event of a new pandemic. Recently, it was shown that preexisting NA-specific antibodies detected in an enzyme-linked immunosorbent assay (ELISA) in a titer of ≥1:40 significantly reduced the duration of seasonal influenza A virus shedding in adults [35]. Age-related differences in NI antibody levels to the pandemic virus H1N1/2009pdm09 were also identified. As was demonstrated, the people born during the circulation of the viruses A/H2N2 and A/H3N2, in the 1960s to 1970s, showed the lowest level of cross-reacting antibodies to the NA of the N1 subtype [24].

Despite the fact that the NA of influenza A/H1N1 and A/H5N1 strains differs in antigenic properties and structure, this protein catalyzes the same enzymatic reaction of the destruction of the α-ketoside bond of N-acetylneuraminic acid. Influenza virus NA is one of the most characterized sialidases; the spatial structure of the NA of several subtypes of influenza A virus and the only subtype of influenza B virus is known, and the mechanisms of the enzyme’s action and the consequences of its inhibition have been well-studied. The active center of the enzyme contains a conservative sequence of 12 amino acids located in the center of each of the four subunits deep in the receptor pocket, the walls and environment of which are also formed by amino acids that are conserved in animal and human influenza A viruses of various subtypes, as well as influenza B viruses. NA has often been seen as a target for antiviral therapy, and has been much less studied in the context of vaccine antigens.

In our study, it was shown that immunization with LAIVs caused a significant increase in antibodies to HA and NA in the seronegative population. The HI and NI antibody titers to A/California/7/09(H1N1)pdm09 influenza virus after natural infection were higher compared with LAIV vaccination.

We demonstrated an increase in the titers of NI antibodies to A/H5N1 virus, not only in patients with an increased level of NI antibodies to the H1N1/2009pdm09 influenza virus, but also when vaccinated with a seasonal LAIV. These data confirm the cross-reactive properties of the NA of the N1 subtype, not only after infection, but also following vaccination with LAIV.

A rather low level of coincidence of the antibody levels to HA and NA in the same serum samples after immunization with LAIV confirmed the previously noted autonomy for HI and NI immune responses when assessing the immunogenicity of LAIV. NI antibodies produced in response to LAIVs vaccination may play an independent protective role [30].

Higher proportions of pre-existing NI antibodies against distant A/H2N2 influenza viruses among elderly volunteers compared with younger people suggests that determining NI antibodies can help identify the most susceptible populations for priority vaccination.

The absence of antibodies to the A/H7N9 influenza virus among people aged 18–49 indicates their susceptibility to infection, and the need for vaccination in the event of a pandemic threat. Despite the fact that, to other viruses, when there were no HI antibodies, a level of pre-existing antibodies to NA was detected, even to the N3 subtype, which had not previously been registered as in circulation, no preexisting NI antibodies against N9 were detected. This may be because of such features of N9 as an additional sialic acid binding site [36]. When vaccinating volunteers with a prototype of A/H7N9 LAIV, the HA of the vaccine strain acted as an immunodominant antigen. However, the low degree of coincidence of the serum immune response to the two surface influenza antigens indicates the need to use ELLA to more fully characterize the immunogenicity of influenza vaccines.

## 5. Conclusions

A number of LAIVs developed against pandemic and potentially pandemic influenza viruses have effectively elicited an immune response in the seronegative population. After double immunization, an increase in inhibition of the hemagglutinating and neuraminidase activity of influenza viruses, as assessed by post-vaccination sera, was revealed. Combining the traditional HI test with the detection of NI antibodies can provide a more complete assessment of the LAIV immunogenicity. NI antibodies to N1 have a certain degree of cross-reactivity against antigenically distant strains. Low preexisting NI antibodies against newly emerged influenza viruses may indicate a high-risk population for priority vaccination.

## Figures and Tables

**Table 1:** The list of clinical trials of live influenza vaccines for use against pandemic and potentially pandemic influenza viruses.

Year	Subtype	Vaccine Strain	“Wild” Type Virus	Genome Composition	Log_10_ EID_50_ per 0.5 mL dose	Age	Ref.
2007	H5N2	A/17/duck/Potsdam/86/92	A/duck/Potsdam/1402–6/86 (H5N2)	7:1	8.3	18–49	[8,9]
2009	H1N1	A/17/California/2009/38	A/California/07/2009 (H1N1)	6:2	7.0	18–60	[10,11]
2012	H7N3	A/17/mallard/Netherlands/00/95	A/mallard/Netherlands/12/00 (H7N3)	6:2	7.0	18–49	[12,13]
2013	H2N2	A/17/California/6/395	A/California/1/1966 (H2N2)	6:2	7.9	18–40	[14]
2014	H7N9	A/17/Anhui/2013/61	A/Anui/1/2013 (H7N9)	6:2	7.5	18–49	[15,16]

**Table 2:** The surface antigen compositions of diagnostic influenza viruses.

Subtype	HA	NA
H7N1	A/horse/Prague/1/56 (H7N7)	A/New Caledonia/20/99(H1N1)
H7N1	A/horse/Prague/1/56 (H7N7)	A/17/California/09/38(H1N1)pdm
H7N2	A/horse/Prague/1/56 (H7N7)	A/California/1/1966(H2N2)
H7N1	A/horse/Prague/1/56 (H7N7)	A/Vietnam/1203/04(H5N1)
H7N2	A/horse/Prague/1/56 (H7N7)	A/Leningrad/134/17/57(H2N2)
H6N1	A/herring gull/Sarma/51c/2006 (H6N1)	A/South Africa/3626/2013 (H1N1)pdm09
H6N9	A/herring gull/Sarma/51c/2006 (H6N1)	A/Anui/1/2013(H7N9)

**Table 3:** The geometric mean titers (GMTs) of hemagglutination inhibition (HI) and neuraminidase-inhibiting (NI) antibodies in vaccinated volunteers after two vaccine doses.

Vaccine Strain	Number of Vaccinated	HI Antibodies (GMT)			NI Antibodies (GMT)		
		Pre-Vaccination	Post-Vaccination	P (Pre/Post)	Pre-Vaccination	Post-Vaccination	P (Pre/Post)
A/17/duck/Potsdam/86/92 (H5N2)	41	4.0	11.4	0.007	6.9	17.1	0.0005
A/17/California/2009/38 (H1N1)	31	6.7	11.4	0.03	13.3	17.2	0.02
A/17/mallard/Netherlands/00/95 (H7N3)	29	2.8	4.7	0.0024	8.8	12.6	0.004
A/17/California/6/395 (H2N2)	27	2.5	5.0	0.007	19.0	35.0	0.02
A/17/Anhui/2013/61 (H7N9)	29	3.0	10.2	0.0001	5.0	6.1	0.043

**Table 4:** Pre-existing NI antibodies against pandemic or potentially pandemic influenza in the sera of non-vaccinated persons.

Year	NA Subtype of Test Virus	Age	Number	% with Antibody Titers > 1:20
2005	A/Vietnam/1203/04(H5N1)	18–20	56	16.0
	A/California/7/09(H1N1)pdm 09	18–20	56	7.1
2006–2007	A/Leningrad/134/57(H2N2)	18–60	60	33.3
2009	A/California/7/09(H1N1)pdm09	18–20	60	30.0
2012	A/mallard/NL/12/00(H7N3)	18–40	39	16.4
2011	A/Viet Nam/1203/04(H5N1)	18–20	26	46.2
2013	A/California/1/66(H2N2)	18–40	36	58.3
		50–84	59	88.1
2014	A/Anhui/1/13(H7N9)	18–49	39	0
2016	A/South Africa/3626/13(H1N1)pdm09	29–81	108	7.0

## References

[1] Krammer F., Fouchier R.A., Eichelberger M.C., Webby R.J., Shaw-Saliba K., Wan H., Wilson P.C., Compans R.W., Skountzou I., Monto A.S. (2018). NAction! How can neuraminidase-based immunity contribute to better influenza virus vaccines?. MBio.

[2] Alexandrova G.I., Budilovsky G.N., Koval T.A., Polezhaev F.I., Garmashova L.M., Ghendon Y.Z., Romanova Y.R., Smorodintsev A.A. (1986). Study of live recombinant cold-adapted influenza bivalent vaccine of type A for use in children: An epidemiological control trial. Vaccine.

[3] Alexandrova G.I., Maassab H.F., Kendal A.P., Medvedeva T.E., Egorov A.Y., Klimov A.I., Cox N.J. (1990). Laboratory properties of cold-adapted influenza B live vaccine strains developed in the US and USSR, and their B/Ann Arbor/1/86 cold-adapted reassortant vaccine candidates. Vaccine.

[4] Small P.A., Cronin B.J. (2017). The Advisory Committee on Immunization Practices’ controversial recommendation against the use of live attenuated influenza vaccine is based on a biased study design that ignores secondary protection. Vaccine.

[5] Negri E., Colombo C., Giordano L., Groth N., Apolone G., La Vecchia C. (2005). Influenza vaccine in healthy children: A meta-analysis. Vaccine.

[6] Kendal A.P. (1997). Cold-adapted live attenuated influenza vaccines developed in Russia: Can they contribute to meeting the needs for influenza control in other countries?. Eur. J. Epidemiol..

[7] WHO Technical Report. Avian Influenza Portfolio. Collected Risk Assessments, Technical Guidance to Public Health Authorities and Advice to the General Public. Stockholm, June 2006. https://ecdc.europa.eu/sites/portal/files/media/en/publications/Publications/0606_TER_Avian_Influenza_Portafolio.pdf.

[8] Lu X., Edwards L.E., Desheva J.A., Nguyen D.C., Rekstin A., Stephenson I., Szretter K., Cox N.J., Rudenko L.G., Klimov A. (2006). Cross-protective immunity in mice induced by live-attenuated or inactivated vaccines against highly pathogenic influenza A (H5N1) viruses. Vaccine.

[9] Rudenko L., Desheva J., Korovkin S., Mironov A., Rekstin A., Grigorieva E., Donina S., Gambaryan A., Katlinsky A. (2008). Safety and immunogenicity of live attenuated influenza reassortant H5 vaccine (phase I-II clinical trials). Influenza Other Respir. Viruses.

[10] Rudenko L., Van Den Bosch H., Kiseleva I., Mironov A., Naikhin A., Larionova N., Bushmenkov D. (2011). Live attenuated pandemic influenza vaccine: Clinical studies on A/17/California/ 2009/38 (H1N1) and licensing of the Russian-developed technology to WHO for pandemic influenza preparedness in developing countries. Vaccine.

[11] Desheva Y., Smolonogina T., Rudenko L. (2011). Detection of anti-neuraminidase antibody in preclinical and clinical studies of live influenza vaccine. Influenza Other Respir. Viruses.

[12] Desheva J., Rudenko L., Rekstin A., Swayne D.E., Cox N.J., Klimov A.I. Development of candidate H7N3 live attenuated cold–adapted influenza vaccine. Proceedings of the International Conference on Options for the Control of Influenza VI.

[13] Rudenko L., Kiseleva I., Naykhin A.N., Erofeeva M., Stukova M., Donina S., Petukhova G., Pisareva M., Krivitskaya V., Grudinin M. (2014). Assessment of human immune responses to H7 avian influenza virus of pandemic potential: Results from a placebo-controlled, randomized double-blind phase I study of live attenuated H7N3 influenza vaccine. PLoS ONE.

[14] Isakova-Sivak I., Stukova M., Erofeeva M., Naykhin A., Donina S., Petukhova G., Kuznetsova V., Kiseleva I., Smolonogina T., Dubrovina I. (2015). H2N2 live attenuated influenza vaccine is safe and immunogenic for healthy adult volunteers. Hum. Vaccin. Immunother..

[15] De Jonge J., Isakova-Sivak I., Van Dijken H., Spijkers S., Mouthaan J., De Jong R., Smolonogina T., Roholl P., Rudenko L. (2016). H7N9 live attenuated influenza vaccine is highly immunogenic, prevents virus replication, and protects against severe bronchopneumonia in ferrets. Mol. Ther..

[16] Rudenko L., Isakova-Sivak I., Naykhin A., Kiseleva I., Stukova M., Erofeeva M., Korenkov D., Matyushenko V., Sparrow E., Kieny M.P. (2016). H7N9 live attenuated influenza vaccine in healthy adults: A randomised, double-blind, placebo-controlled, phase 1 trial. Lancet Infect. Dis..

[17] Harper S.A., Fukuda K., Uyeki T.M., Cox N.J., Bridges C.B. (2005). Prevention and control of influenza: Recommendations of the Advisory Committee on Immunization Practices (ACIP). Morb. Mortal. Wkly. Rep..

[18] Lambré C.R., Terzidis H., Greffard A., Webster R.G. (1990). Measurement of anti-influenza neuraminidase antibody using a peroxidase-linked lectin and microtitre plates coated with natural substrates. J. Immunol. Methods.

[19] Webster R.G., Cox N.J., Stohr K. (2002). WHO Manual on Animal Influenza Diagnosis and Surveillance.

[20] Lindstrom S.E., Cox N.J., Klimov A. (2004). Genetic analysis of human H2N2 and early H3N2 influenza viruses, 1957–1972: Evidence for genetic divergence and multiple reassortment events. Virology.

[21] Chena Z., Kima L., Subbarao K., Jin H. (2012). The 2009 pandemic H1N1 virus induces anti-neuraminidase (NA) antibodies that cross-react with the NA of H5N1 viruses in ferrets. Vaccine.

[22] Sandbulte M.R., Jimenez G.S., Boon A.C., Smith L.R., Treanor J.J., Webby R.J. (2007). Cross-reactive neuraminidase antibodies afford partial protection against H5N1 in mice and are present in unexposed humans. PLoS Med..

[23] Desheva Y., Sychev I., Smolonogina T., Rekstin A., Ilyushina N., Lugovtsev V., Go A., Lerner A. (2018). Anti-neuraminidase antibodies against pandemic A/H1N1 influenza viruses in healthy and influenza-infected individuals. PLoS ONE.

[24] Desheva Y.A., Smolonogina T.A., Donina S.A., Rudenko L.G. (2015). Serum strain-specific or cross-reactive neuraminidase inhibiting antibodies against pandemic A/California/07/2009 (H1N1) influenza in healthy volunteers. BMC Res. Notes.

[25] Monto A.S., Petrie J.G., Cross R.T., Johnson E., Liu M., Zhong W., Levine M., Katz J.M., Ohmit S.E. (2015). Antibody to influenza virus neuraminidase: An independent correlate of protection. J. Infect. Dis..

[26] Marcelin G., Sandbulte M.R., Webby R.J. (2012). Contribution of antibody production against neuraminidase to the protection afforded by influenza vaccines. Rev. Med. Virol..

[27] Bodewes R., Kreijtz J.H., Baas C., Geelhoed-Mieras M.M., de Mutsert G., Van Amerongen G., Van den Brand J.M., Fouchier R.A., Osterhaus A.D., Rimmelzwaan G.F. (2009). Vaccination against human influenza A/H3N2 virus prevents the induction of heterosubtypic immunity against lethal infection with avian influenza A/H5N1 virus. PLoS ONE.

[28] Lai J.C.C., Karunarathna H.M., Wong H.H., Peiris J.S., Nicholls J.M. (2019). Neuraminidase activity and specificity of influenza A virus are influenced by haemagglutinin-receptor binding. Emerg. Microbes Infect..

[29] Monto A., Kendal A. (1973). Effect of neuraminidase antibody on Hong Kong influenza. Lancet.

[30] Memoli M.J., Shaw P.A., Han A., Czajkowski L., Reed S., Athota R., Bristol T., Fargis S., Risos K., Powers J.H. (2016). Evaluation of antihemagglutinin and antineuraminidase antibodies as correlates of protection in an influenza A/H1N1 virus healthy human challenge model. MBio.

[31] Kilbourne E.D., Pokorny B.A., Johansson B., Brett I., Milev Y., Matthews J.T. (2004). Protection of mice with recombinant influenza virus neuraminidase. J. Infect. Dis..

[32] Garten R.J., Davis C.T., Russell C.A., Shu B., Lindstrom S., Balish A., Sessions M., Xu X., Skepner E., Deyde V. (2009). Antigenic and genetic characteristics of swine-origin 2009 A (H1N1) influenza viruses circulating in humans. Science.

[33] Rizzo C., Rota M.C., Bella A., Alfonsi V., Declich S., Caporali M.G., Ranghiasci A., Lapini G., Piccirella S., Salmaso S. (2010). Cross-reactive antibody responses to the 2009 A/H1N1v influenza virus in the Italian population in the pre-pandemic period. Vaccine.

[34] Chen Y.Q., Wohlbold T.J., Zheng N.Y., Huang M., Huang Y., Neu K.E., Lee J., Wan H., Rojas K.T., Kirkpatrick E. (2018). Influenza infection in humans induces broadly cross-reactive and protective neuraminidase-reactive antibodies. Cell.

[35] Maier H.E., Nachbagauer R., Kuan G., Ng S., Lopez R., Sanchez N., Stadlbauer D., Gresh L., Schiller A., Rajabhathor A. (2019). Pre-existing Antineuraminidase Antibodies Are Associated with Shortened Duration of Influenza A (H1N1) pdm Virus Shedding and Illness in Naturally Infected Adults. Clin. Infect. Dis..

[36] Benton D.J., Wharton S.A., Martin S.R., McCauley J.W. (2017). Role of neuraminidase in influenza A (H7N9) virus receptor binding. J. Virol..

